# Prognostic Value of ^18^F-FDG PET/CT-Derived Secondary Lymphoid Organ Ratios and Hematologic Inflammation Markers in Advanced Non-Small Cell Lung Cancer Treated with Nivolumab

**DOI:** 10.3390/jcm15020798

**Published:** 2026-01-19

**Authors:** Erkam Kocaaslan, Ali Kaan Güren, Fırat Akagündüz, Ahmet Demirel, Mustafa Alperen Tunç, Burak Paçacı, Yeşim Ağyol, Pınar Erel, Abdüssamed Çelebi, Selver Işık, Ezgi Çoban, Nazım Can Demircan, Salih Özgüven, Zeynep Ceren Balaban Genç, Nargiz Majidova, Nadiye Sever, Murat Sarı, Osman Köstek, Ibrahim Vedat Bayoğlu

**Affiliations:** 1 Department of Medical Oncology, Marmara University Pendik Training and Research Hospital, 34899 Istanbul, Türkiye; 2 Department of Nuclear Medicine, Marmara University Pendik Training and Research Hospital, 34899 Istanbul, Türkiye; 3 Department of Medical Oncology, VM Medical Park Maltepe Hospital, 34844 Istanbul, Türkiye; 4 Department of Medical Oncology, Haydarpaşa Numune Training and Research Hospital, 34668 Istanbul, Türkiye

**Keywords:** inflammatory metabolic ratios, secondary lymphoid organs, nivolumab, non-small cell lung cancer, prognosis

## Abstract

**Background/Objectives:** This study aimed to evaluate the prognostic value of ^18^F-FDG PET/CT-based secondary lymphoid organ metabolic ratios—spleen/liver (SLR), bone marrow/liver (BLR), and ileocecal region/liver (ILR)—and hematological inflammation markers (neutrophil/lymphocyte ratio [NLR] and systemic immune-inflammation index [SII]) obtained before nivolumab treatment in relation to survival in patients with advanced non-small cell lung cancer (NSCLC). **Methods:** This retrospective single-center study included 79 advanced NSCLC patients who were treated with nivolumab monotherapy at Marmara University Faculty of Medicine Hospital between 2022 and 2024. Pretreatment SLR, BLR, and ILR ratios were calculated from ^18^F-FDG PET/CT examinations; NLR and SII values were obtained from hematological data. Survival outcomes were analyzed using the Kaplan–Meier method, and prognostic factors were assessed using Cox proportional hazards regression analysis. In a subset of patients, an exploratory longitudinal analysis was performed using early follow-up PET/CT to assess follow-up-to-baseline changes in immune-organ metabolic ratios in relation to overall survival. **Results:** High NLR and SII levels were significantly associated with shorter progression-free survival and overall survival. In contrast, no significant associations were observed between PET/CT-derived metabolic ratios (SLR, BLR, and ILR) and survival. Multivariate analysis identified the presence of liver metastases and a high NLR as independent adverse prognostic factors for overall survival. **Conclusions:** In this homogeneous real-world cohort treated exclusively with single-agent nivolumab, PET/CT-derived secondary lymphoid organ metabolic ratios showed limited prognostic value at baseline and during early on-treatment assessment. In contrast, hematological inflammation markers, especially high NLR levels, are strong prognostic indicators of survival and may complement established clinical factors in risk stratification.

## 1. Introduction

Lung cancer is the leading cause of cancer-related deaths worldwide, and approximately 70% of cases are diagnosed at an advanced stage [1]. In recent years, the introduction of immune checkpoint inhibitors (ICIs) into clinical use has resulted in significant improvements in survival outcomes in the treatment of advanced lung cancer [2]. Nivolumab, one of the programmed cell death receptor 1 (PD-1) inhibitors, has become one of the standard treatment options for patients with metastatic non-small cell lung cancer (NSCLC) who have progressed after platinum-based chemotherapy [3].

However, responses to nivolumab treatment are heterogeneous. While some patients achieve durable responses, a significant portion of patients experience early progression or failure to respond to treatment [4]. Therefore, identifying biomarkers that can predict treatment response and long-term survival is of great clinical importance.

Although molecular markers such as PD-L1 expression, tumor mutational burden (TMB), and microsatellite instability (MSI) have been associated with response to immunotherapy, these biomarkers are not always readily available and have limited clinical predictive power [5,6]. Therefore, simple, reproducible, and cost-effective biomarkers are needed.

In recent years, the role of systemic inflammation in tumor progression and immunotherapy response has attracted increasing attention. Hematological parameters, specifically the neutrophil-to-lymphocyte ratio (NLR) and systemic immune-inflammation (SII) index, have been identified as strong indicators of systemic inflammatory status [7,8].

In addition, inflammatory metabolic ratios, spleen/liver standardized uptake value (SUV) ratio (SLR), bone marrow/liver SUV ratio (BLR), and ileocecal region/liver SUV ratio (ILR) obtained from fluorodeoxyglucose (FDG) positron emission tomography/computed tomography (PET/CT) studies are also considered imaging-based indicators of non-tumor immune response. ^18^F-FDG PET/CT can demonstrate not only tumor metabolism but also the activity of the reticuloendothelial system and the metabolic implications of the immune response [9,10,11].

Previous studies have shown an association between increased FDG uptake in the bone marrow or spleen, systemic inflammatory activity, and poor prognosis [9]. However, the prognostic value of these FDG PET/CT-based inflammatory parameters on survival in advanced-stage NSCLC patients receiving nivolumab treatment has been investigated to a limited extent.

This study aimed to evaluate the prognostic significance of pre-treatment ^18^F-FDG PET/CT-derived inflammatory ratios (SLR, BLR, ILR), hematological inflammation markers (NLR, SII), and clinical factors on progression-free survival (PFS) and overall survival (OS) in patients with advanced non-small cell lung cancer treated with nivolumab monotherapy.

## 2. Materials and Methods

### 2.1. Study Design and Participants

This retrospective, single-center study included patients with histopathologically or cytologically confirmed advanced non-small cell lung cancer (NSCLC) who were treated with nivolumab at Marmara University Faculty of Medicine Hospital between January 2022 and December 2024. Inclusion criteria required an ^18^F-FDG PET/CT examination within two months before treatment initiation and a minimum follow-up duration of three months. Patients with controlled brain metastases and a history of previous surgery or thoracic radiotherapy were also included in the study.

Demographic (age, gender, smoking history, body mass index (BMI)), clinical (Eastern Cooperative Oncology Group Performance Status (ECOG) performance score, histological subtype, radiotherapy history, comorbidities, number and location of metastases), and molecular (EGFR, ALK, ROS1, PD-L1) characteristics of the patients were recorded. Patients in the pediatric age group, those with an interval of more than two months between PET/CT scanning and the start of treatment, those with splenic metastases, those with acute or chronic infection or autoimmune diseases, those with other primary malignancies, and those with incomplete clinical or laboratory data were excluded from the study. Additionally, patients who were EGFR, ALK, or ROS1 mutation-positive were excluded from the analysis.

**Figure 1:**
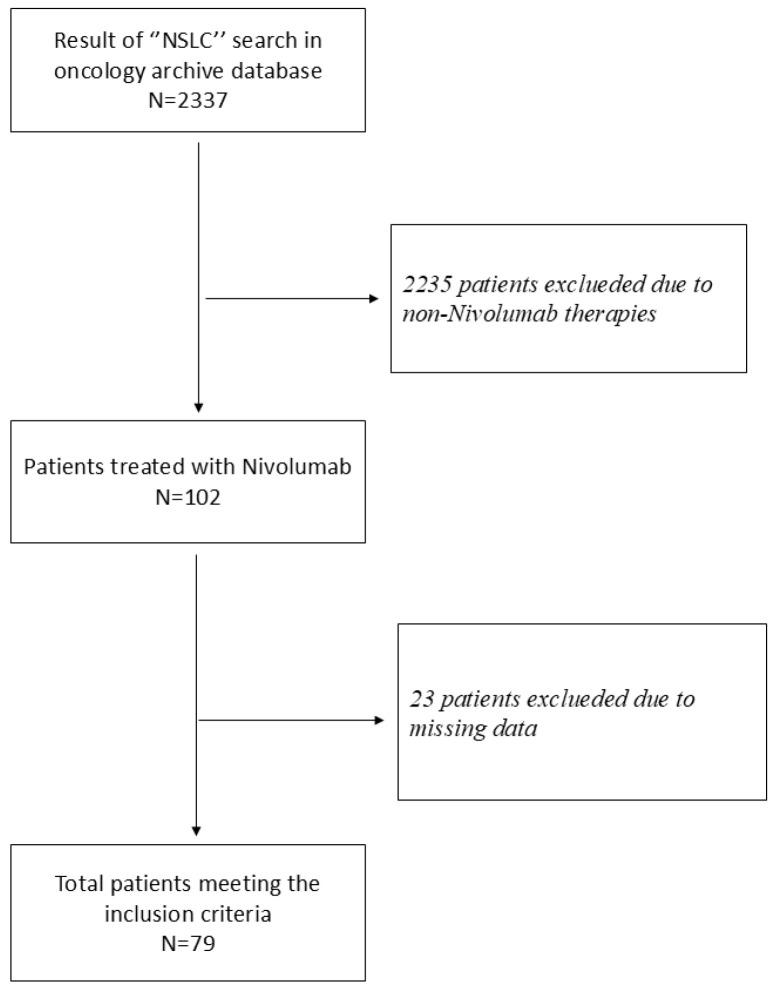
Flow diagram of patient selection.

As a result of the “NSCLC” search performed in the oncology archive database, 2337 patients were identified. 2235 of these patients were excluded because they did not receive immunotherapy. Of the remaining 102 patients, 23 patients with missing data were excluded, resulting in 79 patients being included in the final study population (Figure 1). All patients received nivolumab at a dose of 3 mg/kg, with intravenous infusions every two weeks. Response to treatment was assessed according to iRECIST criteria, with PET/CT performed every 3 months after treatment initiation.

The study was approved by the Clinical Research Ethics Committee of Marmara University Faculty of Medicine (Decision No: 09.2024.1476). All patient data were analyzed anonymously, and the study was conducted in accordance with the Declaration of Helsinki and relevant legal regulations.

### 2.2. Endpoints and Definitions

The primary endpoint was to evaluate the prognostic value of PET/CT-derived secondary lymphoid organ metabolic ratios and hematologic inflammatory markers for OS. The secondary endpoints were to evaluate the association of these PET/CT-based metabolic ratios, hematologic inflammatory markers, and clinical factors with PFS. PFS was defined as the time from the start of immunotherapy to the first date of radiological disease progression or death. OS was defined as the time from the start of treatment to the date of death or last follow-up.

### 2.3. Hematologic and Inflammatory Markers

Systemic inflammatory parameters were calculated using complete blood count results obtained within 14 days before the start of treatment. The neutrophil/lymphocyte ratio (NLR) was calculated as the ratio of the neutrophil count to the lymphocyte count, and the systemic immune-inflammation (SII) index was calculated using the formula (platelets × neutrophils)/lymphocyte. The optimal cut-off value for the SII was determined using receiver operating characteristic (ROC) curve analysis. An SII threshold of 882 was identified as the optimal cut-off (AUC = 0.657, *p* = 0.018). Patients were subsequently dichotomized according to this value. The cut-off value for NLR, commonly used in the literature, was 5 (NLR ≥ 5: high group, NLR < 5: low group) [12].

### 2.4. PET/CT Imaging Protocol

All scans were performed using a Discovery ST PET/CT system (GE Healthcare, Milwaukee, WI, USA). Patients were fasted for at least 6 h before imaging, with only glucose-free water and regular medications permitted. A blood glucose level of <126 mg/dL was required before ^18^F-FDG administration. Each patient received an intravenous injection of ^18^F-FDG at a dose of 5 MBq/kg. Approximately 60 min after tracer injection, a low-dose multi-slice CT scan was acquired using a 16-slice multi-detector scanner (parameters: 80 mA, 140 kV, table speed 27 mm/rotation, slice thickness 3.3 mm) from the upper thighs to the skull base under shallow breathing conditions. Subsequently, a standard whole-body PET scan was performed in 3D mode with an acquisition time of 3 min per bed position (six to eight positions), covering the same anatomical region. PET data were reconstructed using an iterative algorithm, while non-contrast CT images were obtained for attenuation correction. PET images were reconstructed with and without attenuation correction and displayed in axial, coronal, and sagittal planes on an Advantage Windows Workstation using PET Volume Computerized Assisted Reporting (PET VCAR) software (version 4.5).

### 2.5. Image Analysis

A cuboid VOI encompassing the physiological uptake of the liver, spleen, bone marrow, and ileocecal valve was delineated using PET VCAR software, with automatic contouring performed according to a predefined SUV threshold of 42%. SUVmax was defined as the highest voxel intensity within the volume of interest (VOI). A 3 cm diameter spherical VOI was placed in the right hepatic lobe, between the dome and the inferior margin, while carefully excluding central ducts, vascular structures, and metastatic lesions for assessment of the physiological liver SUVmax value. Physiological splenic SUVmax value was assessed by placing a spherical VOI (2–3 cm in diameter, depending on the patient’s spleen size) in the splenic parenchyma, excluding the hilum and major vascular structures. For the evaluation of physiological bone marrow uptake, a spherical VOI with a diameter of 1–2 cm was placed within the L1–L5 vertebral bodies, excluding regions with metastatic disease, infection, or inflammatory involvement, and the SUVmax value was calculated from this area. The physiological ileocecal valve uptake assessment was performed by placing a 3 cm diameter spherical VOI in the ileocecal valve, and the physiological SUVmax value was calculated from this region. The SLR, BLR, and ILR were calculated using SUVmax values obtained from these regions. Since the SLR, BLR, and ILR were not significant in the ROC analysis, they were categorized into two groups based on their median values, which is a commonly used exploratory approach when no validated cut-off exists. All images were reviewed independently by two experienced nuclear medicine physicians.

### 2.6. Statistical Analysis

Statistical analyses were performed using IBM SPSS Statistics for Windows, Version 25.0 (IBM Corp., Armonk, NY, USA). Continuous variables were expressed as mean ± standard deviation (SD), and categorical variables were expressed as number and percentage (%). The normality of continuous variables was assessed using the Shapiro–Wilk test. Since most parameters, including PET/CT-derived metabolic ratios and inflammatory indices, did not show a normal distribution, non-parametric methods were preferred in the analysis. Correlations between PET/CT parameters and hematologic inflammatory markers were evaluated using Spearman’s rank correlation (ρ) coefficient. Survival outcomes were analyzed using the Kaplan–Meier method, and comparisons between groups were performed with the log-rank test. Prognostic factors were examined using univariate and multivariate Cox proportional hazards regression models. A *p* value < 0.05 was considered statistically significant. In addition, an exploratory longitudinal analysis was performed in patients with available early follow-up ^18^F-FDG PET/CT obtained approximately 3 months after nivolumab initiation. Follow-up-to-baseline ratios for spleen-to-liver (SLR), bone marrow-to-liver (BLR), and ileocecal-to-liver (ILR) parameters were calculated using the same acquisition and analysis protocol as baseline imaging. Given the exploratory nature of this analysis and the absence of validated longitudinal cut-off values, patients were dichotomized according to the median value of each ratio. Overall survival was evaluated using Kaplan–Meier analysis and complemented by univariable Cox proportional hazards regression. Multivariable modeling was not performed to avoid overfitting.

## 3. Results

### 3.1. Patient Characteristics

A total of 79 patients were included in the study. The median age was 64 years (range, 42–83), and 88.6% of the patients were male. An ECOG performance status score of 0 was found in 35 patients (44.3%) and ≥1 in 41 patients (51.9%). 13.9% of the patients had received first-line nivolumab therapy, 57% second-line, 19% third-line, and 10.1% fourth-line or higher. A history of smoking was present in most patients (89.9%). Histologically, 59.5% of the cases had non-squamous NSCLC. The proportion of patients with PD-L1 expression was found to be 40.5%. De novo metastatic cases accounted for 60.8%, and those with ≥4 metastatic foci were 44.3%. The most common sites of metastasis were bone (39.2%), brain (27.8%), liver (19%), and adrenal gland (15.2%). A history of thoracic radiotherapy was present in 46.8% of patients (Table 1). Median OS was 10 months for the entire population, while progression-free survival was calculated as 5 months.

### 3.2. Inflammatory Markers

Pre-treatment hematological parameters such as NLR and SII were evaluated as indicators of systemic inflammatory response. In patients with low NLR, median PFS was 5 months (95% CI: 2.96–7.04), compared to 4 months (95% CI: 2.66–5.34) in those with high NLR (*p* = 0.041). According to the cut-off value determined by ROC analysis for SII, median PFS was 5 months (95% CI, 2.94–7.06) in the low SII group and 4 months (95% CI, 2.79–5.21) in the high SII group, showing a highly significant difference (*p* < 0.001). In the overall survival analysis, the prognostic impact of both parameters persisted. For NLR, median OS was not reached in the low NLR group due to the limited number of events, whereas it was 10.0 months (95% CI, 7.98–12.02) in the high group (*p* = 0.024). Regarding SII, median OS was not reached in the low SII group due to the limited number of events, whereas it was 8.0 months (95% CI, 5.81–10.19) in the high SII group (*p* = 0.009). These results demonstrate that increased inflammatory burden is significantly associated with both PFS and OS.

### 3.3. PET/CT Metabolic Parameters and Survival Analysis

**Figure 2:**
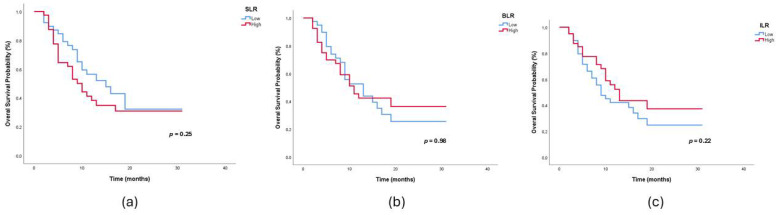
(**a**–**c**) Kaplan–Meier curves according to (**a**) spleen-to-liver ratio (SLR), (**b**) bone marrow-to-liver ratio (BLR), and (**c**) ileocecal-to-liver ratio (ILR).

**Figure 3:**
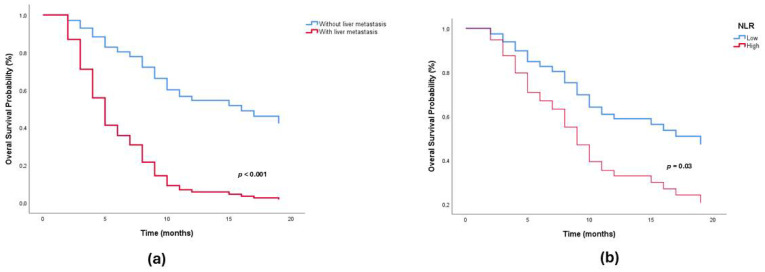
(**a**,**b**) Kaplan–Meier overall survival curves according to (**a**) liver metastasis status and (**b**) neutrophil-to-lymphocyte ratio (NLR) groups.

Secondary lymphoid organ ratios obtained on pre-treatment ^18^F-FDG PET/CT images were defined as SLR, BLR, and ILR. The effects of these parameters on survival were evaluated by the Kaplan–Meier method. PFS and OS analysis results are summarized in Table 2, and overall survival Kaplan–Meier curves are shown in Figure 2a–c. No significant difference was observed between the low and high SLR, BLR, and ILR groups in terms of PFS and OS (*p* > 0.05). These findings suggest that PET/CT-based secondary lymphoid organ metabolism does not play a significant prognostic role in either progression-free or overall survival. In the Cox regression analysis performed to confirm the prognostic value of these parameters, significant variables associated with overall survival were liver metastasis (HR: 4.63, 95% CI: 2.41–8.85; *p* < 0.001), bone metastasis (HR: 1.91, 95% CI: 1.07–3.42; *p* = 0.02), high NLR (HR: 2.04, 95% CI: 1.06–3.91; *p* = 0.03), high SII (HR: 2.16, 95% CI: 1.17–3.97; *p* = 0.01) and ECOG (HR: 2.04, 95% CI: 1.10–3.82; *p* = 0.02). In the multivariate model, only liver metastasis (HR: 4.69; 95% CI: 2.40–9.26; *p* < 0.001) and high NLR (HR: 2.10; 95% CI: 1.04–4.24; *p* = 0.03) remained independent prognostic factors (Figure 3a,b). SII lost significance in multivariate analysis (*p* > 0.05), suggesting its strong correlation with NLR. Although OS was shortened in patients with ECOG ≥1, the difference was not statistically significant (*p* = 0.35). Line of nivolumab therapy (early-line [1st–2nd line] vs. late-line [≥3rd line]) was not significantly associated with overall survival in univariate Cox regression analysis (HR 0.99, 95% CI 0.52–1.88; *p* = 0.98). Similarly, PD-L1 expression (≥1% vs. <1%) was not significantly associated with overall survival in univariate analysis (Table 3).

### 3.4. Relationship Between PET Parameters and Inflammatory Biomarkers

The Shapiro–Wilk test revealed that the variables did not show a normal distribution (*p* < 0.05). Therefore, the relationships between PET parameters and inflammatory indices were evaluated using Spearman’s rank correlation analysis. The relationships between metabolic ratios (SLR, BLR, ILR) obtained from ^18^F-FDG PET/CT and systemic inflammatory indices (NLR and SII) were evaluated using Spearman correlation analysis. A very strong positive correlation was found between NLR and SII (ρ = 0.873, *p* < 0.001). A moderate positive correlation was found between SLR and BLR (ρ = 0.426, *p* < 0.001), and a weak-to-moderate positive correlation was found between SLR and ILR (ρ = 0.302, *p* = 0.007). No significant relationship was observed between the other parameters (*p* > 0.05) (Table 4).

### 3.5. Exploratory Longitudinal PET/CT Analysis

Early follow-up PET/CT was available in 60 patients, depending on the parameter analyzed. Follow-up-to-baseline ratios demonstrated median values of 0.998 for SLR, 1.015 for BLR, and 1.254 for ILR. Kaplan–Meier analyses using median-based stratification revealed no statistically significant differences in overall survival for SLR, BLR, or ILR ratios (log-rank *p* = 0.835, 0.391, and 0.467, respectively). Univariable Cox regression analyses yielded hazard ratios close to unity with wide confidence intervals, confirming the absence of a statistically significant association between longitudinal immune-organ PET/CT ratios and overall survival. Detailed results are presented in Supplementary Table S1.

## 4. Discussion

The most important finding of this study is that pre-treatment ^18^F-FDG PET/CT-derived secondary lymphoid organ ratios SLR, BLR, and ILR obtained before nivolumab treatment in patients with NSCLC were not significantly associated with PFS or OS. It was hypothesized that these ratios, as a reflection of the systemic inflammatory response, could predict immunotherapy efficacy; however, no significant survival advantage was observed in this cohort. Accordingly, PET-derived immune organ metrics may have limited prognostic relevance in this clinical setting and should be interpreted in the context of cohort size and biological complexity. In contrast, the finding that survival was significantly shorter in patients with hematological inflammatory markers, especially the NLR, suggests that the direction of inflammation (protective or suppressive) may play a critical role in determining treatment response. Notably, the inclusion of ILR, which is rarely evaluated in the literature, was intended as an exploratory approach and allowed for the consideration of the metabolic activity of gastrointestinal lymphoid tissues (GALT) related to the immune response and contributed to the more comprehensive evaluation of PET parameters related to secondary lymphoid organs [9,13,14,15]. We acknowledge that ileocecal FDG uptake is physiologically variable and may be influenced by bowel activity, microbiota composition, and local inflammatory processes. Accordingly, ILR was evaluated as an exploratory parameter reflecting gastrointestinal-associated lymphoid tissue activity, and its interpretation should be cautious.

The prognostic value of secondary lymphoid organ ratios (SLR, BLR, and ILR) lies in their potential to reflect a systemic inflammatory response. While inflammation is a physiological indicator of immune system activation, it can also trigger immune suppression in the tumor microenvironment. Therefore, increased FDG uptake carries a dual biological significance, representing either an active immune response to the tumor or chronic tumor-associated inflammation [16]. Previous studies have shown that FDG metabolism, particularly in the spleen and bone marrow, is associated with immune activity, and high FDG uptake in these tissues is often associated with an unfavorable prognosis [9,17]. In contrast, these studies mostly evaluated only SLR or BLR and did not include ILR, which represents the activity of gastrointestinal lymphoid tissues.

PET/CT-based immune organ metrics were hypothesized to provide tissue-level insight into systemic immune activation; however, this signal may be influenced by multiple biological and clinical factors, particularly in real-world retrospective cohorts. In our study, the combined evaluation of these three parameters provided a multidimensional reflection of the systemic immune response. Consistent with the primary findings, Kaplan–Meier and Cox regression analyses revealed no significant differences in PFS or OS for SLR, BLR, and ILR (all *p* > 0.05). Similarly, these PET-based ratios did not independently predict survival in univariate and multivariate Cox analyses. Given the modest sample size and event number, subtle or context-dependent prognostic effects of PET-derived immune organ metrics may not have been detectable in this cohort. However, the significant association of high NLR with OS suggested that hematologic inflammation markers have stronger prognostic value than PET-based indicators in predicting immunotherapy response [18,19]. Importantly, physiologic FDG uptake in secondary lymphoid organs is inherently nonspecific and influenced by multiple factors, including subclinical infection or inflammation, prior systemic therapies, corticosteroid exposure, and tumor-related bone marrow involvement. In particular, bone marrow FDG uptake may be confounded by bone metastases and reactive hematopoiesis, limiting the interpretability of BLR. Although bone marrow measurements were performed by excluding overt metastatic lesions, the presence of bone metastases may still influence global marrow activity and systemic inflammatory signaling, potentially confounding the interpretation of BLR. Similarly, splenic and ileocecal uptake may reflect heterogeneous inflammatory or metabolic processes unrelated to effective antitumor immunity. Consequently, PET-derived immune organ ratios may represent a composite measure of biological signal and noise, diluting potential prognostic associations in small retrospective cohorts.

In the correlation analysis, SLR showed a moderately positive correlation with BLR and ILR, suggesting a metabolic parallelism between secondary lymphoid organs. However, SLR, BLR, and ILR did not show a strong correlation with hematological markers of inflammation (NLR, SII), demonstrating that the relationship between tissue-level metabolic response and peripheral inflammation is complex and not always co-directional. As supported by these correlations, tissue-level metabolic activity and peripheral inflammatory markers may capture distinct and not always overlapping aspects of the host immune response [13].

Previous studies investigating immune-organ ^18^F-FDG uptake during immunotherapy have largely focused on pretreatment PET/CT parameters, while longitudinal data remain limited, particularly in advanced non-small cell lung cancer. Baseline spleen-to-liver and bone marrow-to-liver ratios have been associated with systemic inflammation and survival in heterogeneous cohorts receiving chemotherapy or immunotherapy [9,17]. Longitudinal immune-organ PET/CT assessments have mainly been explored in other disease contexts, such as melanoma, where metabolic changes were related to immune activation or treatment-related inflammatory processes rather than survival outcomes [11,13,20]. In contrast, we evaluated early follow-up-to-baseline immune-organ PET/CT ratios in a single-center cohort treated exclusively with nivolumab. Despite methodological consistency, no significant association between longitudinal SLR, BLR, or ILR ratios and overall survival was observed. This finding suggests that dynamic immune-organ FDG uptake during treatment may be influenced by substantial biological variability and context-dependent factors, limiting its prognostic utility in real-world NSCLC cohorts compared with more robust blood-based inflammatory markers.

Although some studies in the literature have reported that high SLR or BLR is associated with poor prognosis, these studies generally include heterogeneous patient populations receiving combination therapy or in advanced stages [9,17,20]. A similar relationship was not found in our study, which focused on a more homogeneous group of patients receiving single-agent nivolumab therapy. This difference suggests that the prognostic value of PET parameters may vary depending on the type of treatment, the timing of measurement, and the dynamics of the immune response [21].

Liver metastases emerged as a major adverse prognostic factor for overall survival in patients with advanced-stage NSCLC treated with nivolumab. Among the evaluated clinical and biological variables, liver metastases remained a significant independent predictor of poor survival in multivariate analysis. This finding suggests that liver metastases continue to have a negative impact on survival in advanced-stage NSCLC patients undergoing immunotherapy due to both their suppressive effect on the immune microenvironment and their role in altering the systemic inflammatory response. Numerous studies in the literature have emphasized that liver metastases are one of the strongest prognostic indicators that reduce immunotherapy response [22,23]. In our study, an ECOG performance score ≥1 was found to be associated with survival in univariate analysis, supporting the role of overall patient performance in determining treatment response and survival. This is consistent with previous studies reporting that a lower performance score is associated with poorer immune response capacity in NSCLC patients receiving immunotherapy [24]. This variable did not retain statistical significance in the multivariate analysis; this may be due to the ECOG score being associated with stronger prognostic factors such as liver metastases. The negative prognostic impact of bone metastases in univariate analysis may be attributable to increased immune suppressor cells (e.g., myeloid-derived suppressor cells and osteoclast activity) in these patients, in addition to increased tumor burden. This finding is consistent with studies reporting that bone metastases may limit the efficacy of immunotherapy [25].

The significance of the hematological inflammatory markers NLR and SII in univariate analysis supports the impact of systemic inflammation on prognosis. However, only NLR remained independently associated with survival in multivariate analysis. This finding suggests that disruption of the neutrophil–lymphocyte balance may represent the primary determinant of systemic inflammation. This is consistent with studies in the literature reporting that NLR is one of the strongest blood-based prognostic markers for survival in lung cancer patients receiving both chemotherapy and immunotherapy [18,26]. In contrast, the loss of significance of SII in multivariate analysis suggests that the platelet component of this index may have a secondary effect on prognosis. In comparison to imaging-based metrics, NLR directly reflects the balance between innate immune activation and adaptive immune suppression, which is central to immune checkpoint inhibitor efficacy. Its simplicity and lower susceptibility to technical and biological confounders may explain its consistent prognostic performance across different clinical settings.

The specific contribution of the present study lies in the integrated evaluation of PET/CT-derived secondary lymphoid organ metabolic ratios and blood-based inflammatory markers within a relatively homogeneous cohort of advanced NSCLC patients treated exclusively with single-agent nivolumab. Unlike prior studies that included heterogeneous treatment regimens or mixed immune checkpoint inhibitors, this design reduces treatment-related confounding. In addition, the inclusion of the ileocecal-to-liver ratio provides exploratory insight into the metabolic activity of gut-associated lymphoid tissue, which has been rarely addressed in this clinical context. Importantly, the absence of a significant prognostic association for PET/CT-derived immune organ ratios in this setting provides clinically relevant negative evidence, helping to refine expectations regarding the utility of these imaging biomarkers and to prevent their premature adoption without robust validation. In contrast, the consistent prognostic impact of systemic inflammatory markers such as NLR underscores their greater robustness and clinical applicability in this population.

Our study has several limitations. First, the retrospective and single-center design may limit the generalizability of the findings. The relatively small number of patients made it difficult to demonstrate significant associations, particularly for PET parameters. Therefore, the lack of statistically significant associations between PET/CT-derived secondary lymphoid organ ratios and survival outcomes may, at least in part, be related to limited statistical power and the possibility of a type II error rather than the true absence of a biological effect. Accordingly, the negative PET/CT findings should be interpreted as exploratory and hypothesis-generating rather than definitive. Furthermore, ROC analysis was only significant for SII, while other PET parameters were classified according to the median value. Treatment response was assessed only according to PET/CT and iRECIST criteria. Except for PD-L1 expression, all molecular biomarkers that could influence immunotherapy response were not comprehensively examined. Finally, because inflammatory parameters were assessed only before treatment, the prognostic value of dynamic changes during treatment was not considered.

## 5. Conclusions

Our study showed that secondary lymphoid organ ratios (SLR, BLR, and ILR) obtained from pre-treatment ^18^F-FDG PET/CT in advanced-stage NSCLC patients were not significantly associated with PFS or OS in this retrospective single-center cohort. In contrast, hematological inflammatory markers, particularly high NLR and liver metastasis, were identified as independent adverse prognostic factors for survival. These results suggest that PET-based parameters alone may be insufficient for prognostic stratification in this setting and should be interpreted cautiously in conjunction with established clinical and hematological factors. Prospective, multicenter studies with larger cohorts and integrative analytic approaches are warranted to clarify whether PET/CT-derived immune metrics may hold context-dependent or complementary value alongside validated prognostic markers.

## Figures and Tables

**Table 1:** Demographic and clinical characteristics of the patients.

Variable	Value
Age, median (IQR)	64 (42–83)
Gender, male *n* (%)	70 (88.6)
ECOG performance score, *n* (%)	
0	35 (44.3)
1 and above	41 (51.9)
Unknown	3 (3.8)
Treatment line, *n* (%)	
1	11 (13.9)
2	45 (57)
3	15 (19)
4 and later	8 (10.1)
Smoking, *n* (%)	
Current/Former smoker	71 (89.9)
Never	7 (8.9)
Histopathology, non-squamous NSCLC, *n* (%)	47 (59.5)
Prior thoracic RT history, *n* (%)	37 (46.8)
PD-L1 status, 1 and above *n* (%)	32 (40.5)
De novo metastatic, *n* (%)	48 (60.8)
Number of metastatic sites, 4 and above *n* (%)	35 (44.3)
Sites of metastasis, *n* (%)	
Liver	15 (19)
Brain	22 (27.8)
Bone	31 (39.2)
Adrenal	12 (15.2)

ECOG, Eastern Cooperative Oncology Group, RT, Radiotherapy, NSCLC, Non-Small Cell Lung Cancer, PD-L1, Programmed Death-Ligand 1.

**Table 2:** Kaplan–Meier and log-rank analysis of progression-free survival and overall survival according to PET-derived metabolic parameters (SLR, BLR, and ILR).

PET Parameter	Group	Median PFS (Months, 95% CI)	*p* (Log-Rank)	Median OS (Months, 95% CI)	*p* (Log-Rank)
SLR	Low	5.0 (3.0–7.0)	0.408	15.0 (8.5–21.5)	0.253
	High	4.0 (2.5–5.5)		10.0 (7.3–12.7)	
BLR	Low	5.0 (3.7–6.3)	0.499	13.0 (7.0–19.0)	0.985
	High	4.0 (2.2–5.8)		11.0 (7.2–14.8)	
ILR	Low	4.0 (3.1–4.9)	0.497	9.0 (5.5–12.6)	0.227
	High	5.0 (3.5–6.5)		13.0 (10.1–15.9)	

SLR, spleen-to-liver ratio; BLR, bone marrow-to-liver ratio; ILR, ileocecal-to-liver ratio; PET, positron emission tomography.

**Table 3:** Univariable and multivariable Cox proportional hazards regression analyses for overall survival.

	Univariable Analyses			Multivariable Analyses		
	HR	95%CI	*p*	HR	95%CI	*p*
Age, ≥65	1.06	0.60–1.91	0.81			
Gender, female	1.41	0.60–3.34	0.43			
Current smoker	1.56	0.61–3.97	0.35			
BMI ≥ 25	1.59	0.86–2.95	0.14			
ECOG ≥ 1	2.04	1.10–3.82	0.02	1.37	0.7–2.67	0.35
Prior thoracic RT	1.34	0.75–2.40	0.32			
PD-L1 status (≥1)	1.26	0.67–2.37	0.46			
Line of treatment, ≥3	0.99	0.52–1.88	0.98			
De novo metastasis	1.12	0.62–2.04	0.69			
Number of metastatic sites ≥4	1.47	0.83–2.63	0.19			
Liver metastasis	4.63	2.41–8.85	<0.001	4.69	2.40–9.26	<0.001
Bone metastasis	1.91	1.07–3.42	0.02	1.43	0.75–2.72	0.27
Brain metastasis	1.10	0.57–2.14	0.76			
Adrenal metastasis	1.25	0.58–2.68	0.56			
NLR, high	2.04	1.06–3.91	0.03	2.10	1.04–4.24	0.03
SII index, high	2.16	1.17–3.97	0.01	1.42	0.6–3.36	0.42
SLR, high	1.54	0.86–2.75	0.14			
BLR, high	1.01	0.56–1.79	0.98			
ILR, high	1.42	0.79–2.54	0.24			

BMI, body mass index; ECOG, Eastern Cooperative Oncology Group; PD-L1, programmed death-ligand 1; NLR, neutrophil-to-lymphocyte ratio; SII, systemic immune-inflammation; SLR, spleen-to-liver ratio; BLR, bone marrow-to-liver ratio; ILR, ileocecal valve-to-liver ratio.

**Table 4:** Correlation between PET-derived metabolic ratios (SLR, BLR, ILR) and inflammatory biomarkers (NLR, SII).

Variables	ρ (Spearman)	*p*-Value	Interpretation
NLR–SII	0.873	<0.001	Very strong (+)
SLR–BLR	0.426	<0.001	Moderate (+)
SLR–ILR	0.302	0.007	Weak–moderate (+)
SLR–NLR	0.204	0.073	Not significant
SLR–SII	0.182	0.110	Not significant
BLR–ILR	0.153	0.179	Not significant

(+) indicates a positive correlation between the variables. SLR: Spleen-to-liver ratio, BLR: Bone marrow-to-liver ratio, ILR: Ileocecal-to-liver ratio, NLR: neutrophil-to-lymphocyte ratio, SII: Systemic immune-inflammation index, PET: Positron emission tomography, ρ: Spearman’s rank correlation coefficient.

## Data Availability

The data supporting the findings of this study are available from the corresponding author upon reasonable request.

## Supplementary Materials

The following supporting information can be downloaded at: https://www.mdpi.com/article/10.3390/jcm15020798/s1, Table S1: Exploratory longitudinal analysis of immune-organ ^18^F-FDG PET/CT ratios and overall survival.
