# Supplementary material for: Prognostic Value of 18F-FDG PET/CT-Derived Secondary Lymphoid Organ Ratios and Hematologic Inflammation Markers in Advanced Non-Small Cell Lung Cancer Treated with Nivolumab
Source: J Clin Med. 2026 Jan 19;15(2):798. doi: 10.3390/jcm15020798 (PMC12841796; doi:10.3390/jcm15020798)
Supplement: Supplementary file 1 [file jcm-15-00798-s001.zip › jcm-4041695-SI.pdf]

Supplementary Table S1. Exploratory longitudinal analysis of immune-organ ^{18}F-FDG PET/CT ratios and overall survival

Parameter	Group (median)	OS (months)	HR (univariate)	95% CI	*p* value
SLR ratio	< 0.9984	11.0	Reference	–	–
	≥ 0.9984	13.0	0.93	0.49–1.80	0.839
BLR ratio	< 1.0153	16.0	Reference	–	–
	≥ 1.0153	10.0	1.32	0.68–2.57	0.406
ILR ratio	< 1.2542	19.0	Reference	–	–
	≥ 1.2542	11.0	0.79	0.41–1.53	0.481

Early follow-up ^{18}F-FDG PET/CT was available in 60 patients, depending on the parameter analyzed. Ratios were calculated as follow-up-to-baseline values. Analyses were exploratory, and median-based stratification was used to avoid overfitting. HRs were obtained from univariable Cox regression models. Abbreviations: SLR, spleen-to-liver ratio; BLR, bone marrow-to-liver ratio; ILR, ileocecal-to-liver ratio; FDG, fluorodeoxyglucose; PET/CT, positron emission tomography/computed tomography; OS, overall survival; HR, hazard ratio; CI, confidence interval.
